# Mindful moms: acceptability and impact of co-designed and digitally delivered video meditations for pregnant and parenting women with opioid use disorder

**DOI:** 10.1080/07853890.2025.2486585

**Published:** 2025-04-18

**Authors:** Sarah E. Lord, Deepika Rao

**Affiliations:** ^a^ Center for Technology and Behavioral Health, Geisel School of Medicine at Dartmouth College, Hanover, NH, USA; ^b^ Department of Biomedical Data Science, Geisel School of Medicine at Dartmouth College, Hanover, NH, USA; ^c^ Department of Psychiatry, Dartmouth Health, Lebanon, NH, USA

**Keywords:** Mindfulness, meditation, digital delivery, pregnant and parenting women, opioid use disorder, stress

## Abstract

**Introduction:**

Perinatal opioid use disorder (OUD) remains a public health epidemic. Stress, anxiety and depression are disproportionately high among this population and are associated with poor recovery outcomes. Mindfulness interventions show promise for supporting recovery for women. This paper reports results of a pilot study to evaluate initial efficacy and acceptability of digitally delivered mindfulness meditation videos to reduce stress and promote mindfulness among women in recovery.

**Methods:**

Women with lived experience of OUD were recruited from three outpatient programs that provided care to pregnant and parenting women with a history of opioid use in rural northern New England (2 maternity care settings that offered buprenorphine as part of their service menu and 1 academic substance use treatment setting). In a pre-post study design, participants were randomly assigned to receive four of 16 short meditation videos, each delivered by email in a survey link over a 2-week period (2 per week) Videos were co-designed in earlier work with representative end-users, guided by evidence-based mindfulness interventions. Assessment included the Perceived Stress Scale and the Mindfulness Attention Awareness Scale. Participants rated each video on usefulness, enjoyability, ability to lower anxiety, and intention to use in the future. Participants also provided open-ended feedback about the videos. Data were analyzed using descriptive statistics, paired t-tests, and generalized linear modeling.

**Results:**

A total of 20 women, ages 24–36 years, completed the pilot study. Most participants (95%) were white and non-Hispanic, reflecting the rural region. Marginal mean perceived stress scores decreased significantly from 21.49 to 19.85 [*p* = 0.05, *d* = 0.43] and mean mindfulness scores increased significantly from 3.47 to 3.76 [*p* = 0.04, *d* = 0.45]. Overall, the meditation videos were rated as highly acceptable and useful and a majority (80%) indicated intention to use the meditations in the future.

**Conclusion:**

Digitally delivered meditation videos were highly acceptable and useful to participants and the low dose intervention reduced stress and improved mindfulness. Findings inform directions for future research with larger samples to evaluate the effectiveness of this accessible digital intervention to support women in recovery and strategies for broadly implementing the intervention.

## KEY MESSAGES

- A low dose of a digitally delivered meditation video intervention significantly reduced perceived stress and improved mindfulness in women with opioid use disorder.
- The meditation videos were highly acceptable and useful to participants and the digital implementation was demonstrated feasible.
- Participant feedback informed further refinements of meditation videos to promote engagement.
- The pilot study seeds a strong foundation from which to conduct larger, fully powered randomized controlled studies to evaluate effectiveness and implementation of a digital delivered meditation intervention to support recovery for women with opioid use disorder.

## Introduction

Opioid use among pregnant and parenting women continues to be a national public health epidemic. Current standard of care for treatment of OUD is medication treatment, most often with methadone or buprenorphine [1]. Even when treatment is initiated, retention in treatment is low, decreasing most precipitously during the early postpartum period [2–6].

Mental health conditions, trauma, and stressful social circumstances can increase relapse risk. Women with OUD have high rates of co-occurring mental health conditions, with depression and anxiety conditions ranging as high as 65–75% [7–9]. Exposure to physical, sexual, and emotional trauma is also common among women with substance use conditions [10]. Additionally, roughly 78% of women with OUD are also diagnosed with at least one non-opioid substance use disorder [9].

Mindfulness-based interventions have demonstrated promise for supporting recovery and improving mental health for persons with substance use conditions (e.g. Refs. [11–21]). Meta-analyses of mindfulness intervention studies indicate small-to-large effects of mindfulness interventions in reducing frequency and severity of substance use, intensity of drug craving and severity of experienced stress, and improving mental health [22–24]. Studies demonstrate these positive effects for women with substance use conditions. For example, mindful awareness body touch-therapy (MABT) as an adjunct to substance use treatment for women yielded moderate to large effects, including significantly fewer substance use days, reduced craving, and improvement in depression, anxiety, dissociation, and perceived stress compared to treatment as usual [19,20]. A study of mindfulness-based relapse prevention (MBRP) with women in a residential treatment center yielded significantly fewer drug use days and fewer legal and medical problems at 3 month follow up relative to standard of care relapse prevention [21]. A 12-week mindfulness-based parenting intervention for women in treatment for opioid use disorder resulted in significant pre-post improvements in quality of parenting behaviors [25], and significant decreases in depression [26]. A 6-week mindfulness in pregnancy intervention for women at risk yielded reductions in perceived stress, pregnancy specific stress, anxiety, and depression and increases in mindfulness that were sustained at 7 months post intervention [27]. Evidence indicates that mindfulness interventions work through reducing stress and craving and improving mindfulness and interoceptive awareness [15,19,20,28–32].

Mindfulness interventions promote awareness of breath and breathing, thoughts, emotions, and body sensations. Non-judgment and acceptance of momentary experiences are central to mindfulness approaches, as is an emphasis on compassion for others and for oneself. Meditation is a key component of mindfulness interventions. Although mindfulness interventions have demonstrated benefit, these manual-based interventions can be time and labor intensive (e.g. 8 weeks/2 h per week). In addition, these interventions are largely delivered in person, creating barriers to access, broad uptake, and ongoing engagement. Digital delivery of mindfulness interventions can address these barriers by offering low cost, broad accessibility of interventions that can be delivered with high fidelity by nature of technology platforms. There is emerging evidence for smartphone-based mindfulness training approaches for tobacco use (e.g. Refs. [33,34]). The purpose of this pilot study was to evaluate initial efficacy of a brief, digitally delivered video-based mindfulness meditation intervention on perceived stress and mindfulness of pregnant and parenting women with opioid use disorder. We also assessed feasibility and acceptability of the digitally delivered meditation video by participants.

## Methods

All study procedures were approved by the Committee for Protection of Human Subjects (CPHS) Institutional Review Board at the authors’ institution (STUDY00031364). The study adheres to the Declaration of Helsinki.

### Recruitment

Pregnant and parenting women aged 18 and older who were in treatment for an opioid use disorder were recruited from three outpatient settings in northern New England. Two of the settings were maternity care practices that provided buprenorphine for OUD as part of the service menu; the third was an academic outpatient substance use treatment program. Recruitment occurred through clinicians providing study flyers to patients/clients and flyers posted in high patient traffic areas. Clinicians at study sites were informed about the study through introductory meetings with the study team. Patients/clients who expressed interest in the study by contacting study staff were sent an information sheet about the study.

### Informed consent

Those who wished to participate were verbally consented over the phone by study research staff. Verbally consented participants were then emailed a secured online survey *via* SurveyMonkey. The survey included two eligibility questions (Are you a pregnant or parenting woman aged 18 years or over? Are you living with a current or past opioid use condition?) and a digital consent to read through and indicate consent *via* check-box approval. Upon documentation of consent, participants received the online baseline assessment survey *via* email. Thus, both verbal and documented consent were obtained from participants for the pilot.

### Guided meditation videos

Sixteen guided meditation videos were developed as part of a web-based digital companion for a planned yoga-mindfulness intervention study. The foundation content of the meditations was based on Mindfulness Based Relapse Prevention [11]. Meditation content included focus on building awareness of sensations in the body (3), awareness of breath and breathing (3), awareness of sounds and sights (2), awareness of thoughts (1), attention (4), urge surfing (1), and loving kindness and compassion (2). The content was further informed during formative focus group work as part of a larger parent yoga-mindfulness study. A sample of 23 pregnant and parenting women receiving treatment for opioid use shared their experiences with yoga and meditation, described barriers and facilitators to participating in mindfulness interventions, and discussed their needs regarding digital mindfulness approaches (manuscript in preparation). Themes from these discussions informed the content, imagery, voice and presentation style of the meditations. The meditation content and delivery approach were further informed by women with lived experience who participated in live piloting sessions of the parent study yoga-mindfulness intervention, of which the meditations were a part.

Revised scripts were audio recorded by a member of the study team using an Olympus LS-100 audio recorder in a soundproof room at the authors’ institution. The recorded audio was edited by a member of the study team using Adobe Premiere Pro and Adobe Audition. Rotating stock images that matched the topics of the meditations were used as visuals. Each meditation was developed to be no more than 3 min. Finalized videos were uploaded to Vimeo and embedded into SurveyMonkey online surveys. Each participant was randomly assigned to receive four of the 16 guided meditation videos (i.e. total full dose approximately 12 min).

### Meditation video intervention delivery

After completion of baseline, participants were sent by email a unique online survey link that included an assigned meditation video twice a week for two consecutive weeks (i.e. 2 meditation videos per week). Each unique link was coded with the participants non-identifying study identification number. After viewing a video meditation, participants were automatically taken in the survey to a brief series of closed-ended questions to rate the meditation on acceptability, usefulness, and ability to reduce anxiety, as well as their intention to use the video meditation in the future. An open-ended question elicited participants feedback on parts of the video meditation most and least liked. At the end of the two-week period, all participants were sent a unique link to the post-intervention assessment survey.

### Measures

The pre- and post-assessment surveys included frequently used and psychometrically sound indicators of stress and mindfulness for the target population. *Stress* was measured with the 10-item Perceived Stress Scale (PSS) [35]. Items are rated on a 5-point scale (0 = never, 1 = almost never, 2 = sometimes, 3 = fairly often 4 = very often), with a total score ranging between 0 and 40. Scores of 0–13 indicate low stress, 14–26 indicate moderate stress, and 27–40 indicate high perceived stress. *Mindfulness* was measured with the Mindfulness Attention Awareness Scale (MAAS), a 15-item scale designed to assess a core characteristic of mindfulness: being present in awareness and attention [36]. Items are rated on a 6-point scale (1 = almost always, 2 = very frequently, 3 = somewhat frequently, 4 = somewhat infrequently, 5 = very infrequently, 6 = almost never). A total scale score was created as the mean of the items. Both measures have been extensively used in other mindfulness studies with similar populations [37–40]. The post-intervention survey also included participant sociodemographic questions.

### Post video acceptability and feedback survey

After reviewing each guided meditation, participants were asked to rate the video for usefulness (“I think the meditation video could be useful in my life”), enjoyability (“I enjoyed watching the meditation video”), ability to lower anxiety (“Watching the meditation video lowered my anxiety”), pacing, and intention to use the meditation in the future (“I would watch more meditation videos like this one”). Usefulness, enjoyability, and intention to use in the future were rated on a 7-point scale (1 = strongly disagree, 7 = strongly agree) and pacing was rated on a 5-point scale (1 = way too slow, 5 = way too fast). To gather qualitative feedback on the videos, participants were also given the opportunity to respond to 3 open-ended questions: “What did you like about the meditation video?”, “What didn’t you like?”, and “What would you change about the meditation video to make it more enjoyable or useful?”. Participants were compensated with gift cards for completing the baseline assessment and two-week post assessment (baseline: $10;post assessment: $10) and $10 for each video meditation used (total: up to $60 in gift cards).

### Data analysis

Quantitative data were analyzed using SPSS Version 28. Descriptive statistics were used to examine participant sociodemographic characteristics of participants and sample distributions of outcome indicators. We tested residual distributions for normality for both outcomes using standard procedures [41]. Tests indicated that pre-post perceived stress residuals were not normally distributed; mindfulness residuals were normally distributed. Since GEE does not assume normality, we proceeded with use of this analysis approach. We conducted generalized linear modeling analyses (using estimating equations) to examine the effect of the intervention on perceived stress and mindfulness scores. This approach is consistent with other mindfulness intervention studies with a single sample and repeated measures [25,27]. To examine the relative impact of individual items for each outcome, items for each outcome measure were compared pre- and post-intervention *via* paired t-tests and Cohen’s D effect sizes are reported.

Quantitative acceptability ratings of each video were analyzed descriptively with means and standard deviations and categorically as percentages. Responses to the open-ended questions were analyzed using inductive content analysis using open coding. A study team member (DR) who was not involved in data collection independently created an initial codebook with major thematic codes. The categories were reviewed and discussed with the full team for accuracy and completeness and the final refined code book was approved by the first author (SEL). The data were coded by DR and two research assistants using the code book. The final categories were reviewed and discussed with the full team for accuracy and completeness and a final list of qualitative categories was created by SEL and DR. Qualitative data were transformed into quantitative frequencies to identify the most salient participant perspectives.

## Results

### Sample characteristics

Twenty-five eligible women consented to the study and completed the pre-intervention baseline assessment survey. Twenty participants (80%) completed the video meditation interventions and the post-intervention survey. As depicted in Table 1, the mean participant age of the final sample was 30.05 ± 3.93 years (range 24–36 years). Most participants (95%) were white and non-Hispanic. Thirty percent had high school or equivalent level of education. Most participants (80%) were not partnered. Sixty percent had some education post high school, and 10% had a college degree. Fifteen percent of participants were employed, and most were receiving public assistance or benefits (90%).

**Table 1.** Sociodemographic characteristics.

Variables (*n* = 20)	Mean (*SD*)
Participant Age	30.05 (3.93)
	Frequency *n* (%)
Race	
White	19 (95%)
American Indian/Alaska Native	1 (5%)
Hispanic	1 (5%)
Marital Status	
Single	16 (80%)
Partnered	3 (15%)
Preferred not to answer	1 (5%)
Education	
High School/GED	6 (30%)
Some college (no degree)	12 (60%)
Associate or Bachelor’s degree	2 (10%)
Employment status	
Employed full-time	3 (15%)
Unemployed	8 (40%)
Full-time Homemaker	9 (45%)
Insurance	
Medicaid/Medicare/Public	18 (90%)
Uninsured	1 (5%)
Missing	1 (5%)

### Perceived stress

Pre-post estimated marginal and parameter estimates for perceived stress are summarized in Table 2. There was a significant decrease in mean perceived stress scores from 21.49 to 19.85 with a moderate effect size [*W* = 3.85, *p* = 0.05, *d*=.43]. Item-level perceived stress results are shown in Table 3. On average, the sample reported moderate stress. More individuals were in the “low stress” category post intervention (20%) as compared to baseline (5%). There was a statistically significant decrease in item-level scores for item 3 (…felt nervous or stressed?) from 2.95 to 2.7 (*t* = 1.75, *p* < 0.05), item 6 (“…could not cope with all the things you had to do?”) from 2.2 to 1.6 (*t* = 3.94, *p* < 0.001), and item 8 (“.not feeling on top of things”) from 1.95 to 1.65 (*t* = 1.82, *p* < 0.05) indicating decreased perceived stress and increased ability to cope with stress.

**Table 2.** Pre-post marginal means and parameter estimates for perceived stress and mindfulness scores.

	Perceived Stress		Mindfulness	
Pre-Survey Mean (SE)	21.49 (1.20)		3.47 (0.17)	
Post-Survey Mean (SE)	19.85 (1.36)		3.76 (0.17)	
Cohen’s d Effect Size	0.43		0.45	
Parameters	B (SE)	*P*	B (SE)	*P*
Intercept	21.49 (1.20)	0.00	3.47 (0.17)	0.00
Time	−1.64 (0.84)	0.05	0.29 (0.14)	0.04

**Table 3.** Change in item-level perceived stress ratings (*n* = 20).

	Pre-Survey Mean (*SD*)	Post-Survey Mean (*SD*)	Cohen’s *d* Effect Size
Stress Categories *n* (%)			
Low Stress	1 (5%)	4 (20%)	
Moderate Stress	15 (75%)	13 (65%)	
High Stress	4 (20%)	3 (15%)	
Item 1 – upset by unexpected things	2.35 (0.87)	2.50 (0.95)	0.20
Item 2 – unable to control important things	2.30 (0.86)	2.20 (0.95)	−0.14
Item 3 – nervous or stressed	2.95 (0.76)	2.70 (0.92)*	−0.39
Item 4 – confident handling problem^#^	1.55 (0.83)	1.30 (0.80)	−0.31
Item 5 – things going your way^#^	1.89 (0.91)	1.95 (0.89)	0.06
Item 6 – not cope with all the things	2.20 (0.89)	1.60 (0.68)**	−0.88
Item 7 – control irritations^#^	2.15 (0.75)	1.80 (0.83)	−0.05
Item 8 – on top of things^#^	1.95 (0.76)	1.65 (0.75)*	−0.41
Item 9 – angered by things	2.30 (0.92)	2.35 (0.81)	0.08
Item 10 – could not overcome difficulties	2.15 (0.93)	1.80 (1.0)	−0.32

* One-sided *p* < 0.05 for paired *t* test.

** Two-sided *p* < 0.01 for paired *t* test.

^#^ Items were reverse scored for all calculations.

### Mindfulness

There was a significant increase in mindfulness scores from post intervention from 3.47 to 3.76 [*W* = 4.22, *p* = 0.04, *d* = 0.45] (see Table 2), representing a moderate effect size. Item-level statistics are reported in Table 4. There were statistically significant improvements in scores for item 2 (“…break or spill things) from 3.9 to 4.40 (*t* = 1.75, *p* < 0.05), item 8 (“I rush through activities without being really attentive to them.”) from 3.1 to 3.8 (*t* = 2.67, *p* < 0.01), and item 10 (“I do jobs or tasks automatically, without being aware of what I’m doing.”) 3.45 to 4.05 (*t* = 2.35, *p* < 0.05).

**Table 4.** Changes in mindfulness scores (*n* = 20).

	Pre-Survey Mean (*SD*)	Post-Survey Mean (*SD*)	Cohen’s *d* Effect Size
Item 1 – not conscious of emotion	3.60 (1.14)	3.65 (1.18)	0.04
Item 2 – break or spill things	3.90 (1.45)	4.40 (1.19)*	0.39
Item 3 – difficult to stay focused on present	3.65 (1.50)	3.90 (1.25)	0.19
Item 4 – walk quickly without paying attention	2.80 (1.44)	2.95 (1.36)	0.09
Item 5 – not notice tension or discomfort	3.50 (1.10)	3.95 (1.19)	0.34
Item 6 – forget a person’s name immediately	3.15 (1.76)	3.20 (2.02)	0.04
Item 7 – running on automatic	3.55 (1.28)	3.50 (1.36)	−0.03
Item 8 – rush through activities	3.10 (1.29)	3.80 (1.5)*	0.60
Item 9 – lose touch when focused on goal	3.50 (1.19)	3.85 (1.14)	0.22
Item 10 – do jobs/tasks automatically	3.45 (1.32)	4.05 (1.15)*	0.53
Item 11 – listen to someone while doing something	2.25 (1.25)	2.65 (1.14)	0.57
Item 12 – drive on automatic pilot	4.75 (1.48)	4.70 (1.45)	−0.04
Item 13 – preoccupied with future and past	2.75 (1.21)	3.05 (1.32)	0.24
Item 14 – do things without paying attention	3.40 (1.35)	3.65 (1.18)	0.22
Item 15 – snack without awareness	4.65 (1.14)	5.05 (1.10)	0.33

* One-sided *p* < 0.05 for paired *t* test; ^a^Mindfulness Attention Awareness Scale.

### Video meditation acceptability

Each guided meditation video was viewed by 5 participants (i.e. *n* = 80 responses for the 16 videos). Each of the 20 participants used all four of the meditations randomly assigned to them, reflecting good feasibility of the email delivery strategy of the meditation videos and good overall engagement with the videos. As summarized in Table 5, the meditations received high ratings of acceptability, with over 80% agreeing that the guided meditations were useful, enjoyable, lowered their anxiety, and that they intended to watch more meditation videos in the future.

**Table 5.** Video meditation acceptability ratings (1 = strongly disagree, 7 = strongly agree).

	Mean (*SD*)	Agree *n* (%)	Neutral *n* (%)	Disagree *n* (%)
Useful (Averaged) (*n* = 80)	5.66 (1.22)	67 (83.8%)	10 (12.5%)	3 (3.7%)
Lowered Anxiety (Averaged) (*n* = 80)	5.68 (1.34)	65 (81.3%)	10 (12.5%)	5 (6.2%)
Enjoyable (Averaged) (*n* = 80)	5.68 (1.35)	65 (81.3%)	10 (12.5%)	5 (6.2%)
Intention to Use (Averaged) (*n* = 80)	5.53 (1.37)	64 (80%)	7 (8.8%)	9 (11.3%)

The three open-ended questions were coded for content themes (Table 6). The most frequent feedback again reflected an overall positive assessment of the videos (58 mentions, with 15/20 (75%) of participants mentioning they liked/enjoyed the videos overall). Positive descriptions of specific video features were the second-most frequent feedback category, with 41 mentions and 17/20 (85%) of participants describing video features and content positively. Most participants also re-iterated positive outcomes, including lowered anxiety and stress, increased focus and attention, calmed mind, and relaxed body (37 mentions, with 16/20 (80%) of participants reporting positive outcome experiences). Five participants (25%) reported that the meditations validated their feelings and helped with cravings and recommended that others use the meditations. Feedback for improvements related to features of the video meditations, such as duration, pace, sounds, and music (51 mentions with 18 (90%) describing potential improvements, most of which were later incorporated by the study team. Participants (55%) also recommended changes to content based on personal preferences, which were not consistent across the sample. See Table 6 for sample quotes.

**Table 6.** Qualitative feedback categories (frequency of mentions).

Qualitative Categories	Overall Frequency of Mentions	Unique Mentions	Representative Quotes
General Overall Positive: Nothing to change/Disliked nothing/Liked/ Enjoyed/ Pleasantly Surprised	58	15	*“I do not dislike anything. This meditation, like all of these meditations have been great. Esp for people new to meditation!”* – Pt 5 *“I liked the entire video very much.”* – Pt 10 *“I enjoyed it.”* - Pt 9
Positive descriptions of meditation video features, e.g. content visuals, guide’s voice	41	17	*“I liked being able to imagine what a certain situation would make me feel like emotionally.” –* Pt 2 *“I liked the woman’s voice. It was calming.”* – Pt 20
Positive Outcomes from using the meditations: e.g. lower anxiety, increased self-focus & attention, calming, relaxing	37	16	*“It helped calm my mind and helped my anxiety” –* Pt 6 *“Meditation brings me focus, as well as grounds me. I feel more capable and relaxed after finishing.”* – Pt 18
Positive Experiences: Meditations validate feelings and thoughts, face urges	8	5	*“I conquered my fears and I felt like I was in charge of my cravings.”* – Pt 10
Recommend meditations for others in recovery	6	5	*“This is something that should be just taught in any kind of situation - recovery, mental health, high blood pressure issues and so many more!”* – Pt 8
Areas of Improvement: Meditation Video Characteristics e.g. duration of videos, pace of content, added features (music), sounds	51	18	*“It was too slow paced for me and I got distracted easily. My mind wandered.”-* Pt 3 *“Maybe add some low, soft meditation music in the background.”* – Pt 12
Changes based on personal preferences of content	31	11	*“I did not like picturing myself in a triggering scenario as I feel I deal with stress enough in my every day life.”* – Pt 2 *“I prefer meditations that are not guided.”* – Pt 14

## Discussion

In this pilot study we demonstrated that a low dose (approximately 12 min) of a digitally delivered meditation video intervention delivered over a two-week period significantly reduced perceived stress and improved mindfulness and attentional awareness among women with OUD. Participants indicated high acceptability of each of the meditation videos and provided positive feedback about areas for improvement, largely related to enhancing the features of the video meditations. Our findings also provide support for the feasibility of delivering the meditation video intervention to this vulnerable rural population by way of a simple, widely accessible digital platform. Overall, these results indicate strong potential for the mindfulness meditation intervention to support recovery for women with OUD.

Our findings are consistent with other studies that have demonstrated the positive impact of mindfulness interventions on stress and mindfulness constructs [12,15,16,19,20,25–27,30,32]. Stress is strongly associated with increased craving for opioids, leading to increased use and decreased treatment retention [42]. Increased mindfulness is proposed to alleviate cue-induced craving, increase top-down control, help emotional regulation, and reduce distress among individuals who use substances [43,44].

The high acceptability and feasibility of the simple email delivery of the meditation videos reflects the growing recognition of the power of digital platforms for delivery of evidence-based mindfulness interventions [14,33,34,45,46]. Digital platforms can overcome many of the barriers to traditional intervention delivery, including participant engagement and delivery fidelity. The high acceptability ratings, including intentions to use in the future, are promising results since intention to use an intervention is a close proxy for actual future use [47]. Future research is needed to evaluate the range of digital implementation strategies (e.g. smartphone apps, text messaging, email) that can be used to deliver effective mindfulness interventions directly to individuals when they need them the most. There is exciting opportunity to advance the field in this arena.

The input of women with lived experience in the creation of the meditation videos contributed to the success of our approach. We also received valuable feedback from the pilot study participants to further improve the features of the meditation videos to promote participant engagement. Involvement of end-user stakeholders in co-creation of interventions can lead to interventions that are perceived as relevant and engaging, producing more effective and sustained implementation of mindfulness interventions.

### Limitations

This was a pilot study conducted with a small convenience relatively homogenous sample. Future research should involve more diverse samples to achieve more generalizable findings. While our findings are not generalizable, the promising results offer critical data to inform a larger study to evaluate effectiveness and implementation of the full mindfulness meditation intervention in future research. Larger, fully powered randomized controlled trials are needed to evaluate minimum dose for longer-term impacts, mechanisms of effect, and optimal dissemination and implementation strategies. For example, the intervention could be evaluated as a standalone intervention, or as an adjunct to other care (e.g. medication treatment, group counseling). Depending on the study design, larger studies will consist of sample sizes ranging anywhere from 120 to 300 or more participants. While the objective of our study was to evaluate initial impact of the intervention on perceived stress and mindfulness, our qualitative findings suggest participants experienced a reduction in anxiety symptoms and an increase in control of cravings. Future research will include evaluation of the effect of the digitally delivered mindfulness intervention on mental health and substance use outcomes.

## Conclusion

Pregnant and parenting women with lived experience of OUD found the low dose of guided meditation videos useful, enjoyable, and effective. The meditation videos reduced stress and increased mindfulness over the span of 2 weeks. Participant feedback was invaluable in co-designing a digitally delivered video-based mindfulness intervention that is engaging and perceived as relevant for this target population and can be readily tested in larger studies. This digital mindfulness intervention can contribute to the growing science and evidence surrounding the promise of mindfulness interventions as powerful nonpharmacologic approaches to substance use and related conditions [48].

## Acknowledgements

We want to express our deep gratitude to the study participants, our best teachers. We would also like to thank Ashley Maher for her support in creation of videos and data collection and Sena Park and Stephanie Langlois for their contributions to data analysis. We would also like to acknowledge Holle Black for lending her expertise in trauma-informed mindfulness and for her help with recording intervention videos. Portions of the work in this manuscript were presented at the 2019 (Park City, Utah) and 2021 (virtual) Addiction Health Services Research Conference meetings.

## Data Availability

The authors agree to make data and materials supporting the results or analyses presented in this paper available upon reasonable request.
